# Adolescent With Round Opacity

**DOI:** 10.1016/j.acepjo.2026.100531

**Published:** 2026-10-26

**Authors:** Taketo Baba

**Affiliations:** Department of Pediatrics, Division of Emergency Medicine, University of Texas Southwestern, Children’s Medical Center, Dallas, Texas, USA

**Keywords:** pediatric lung abscess, POCUS, pediatrics

## 1 Case Presentation

A healthy 16-year-old adolescent boy presented to the pediatric emergency department with a 2-week history of fever, worsening productive cough with brown sputum, and right-sided pleuritic chest pain for 5 days. Physical examination revealed a fatigued-appearing young adolescent boy with right-sided rhonchi. Vital signs were stable (temperature 37.2 °C, oxygen saturation 98% on room air).

An initial chest radiograph was interpreted by a pediatric radiologist as a right upper lobe round opacity, consistent with round pneumonia (Fig 1). An air-fluid level within the round opacity went unreported on the initial radiograph reading. Lung point-of-care ultrasound performed by the emergency physician revealed an irregularly shaped, heterogeneous, hypoechoic lesion demonstrating posterior acoustic enhancement and internal echogenic debris moving with respiration (Fig 2; Video 1, Video 2). Subsequent chest computed tomography (CT) confirmed a 4.8 cm cavitary lesion in the right upper lobe (Fig 3).

**Figure 1:**
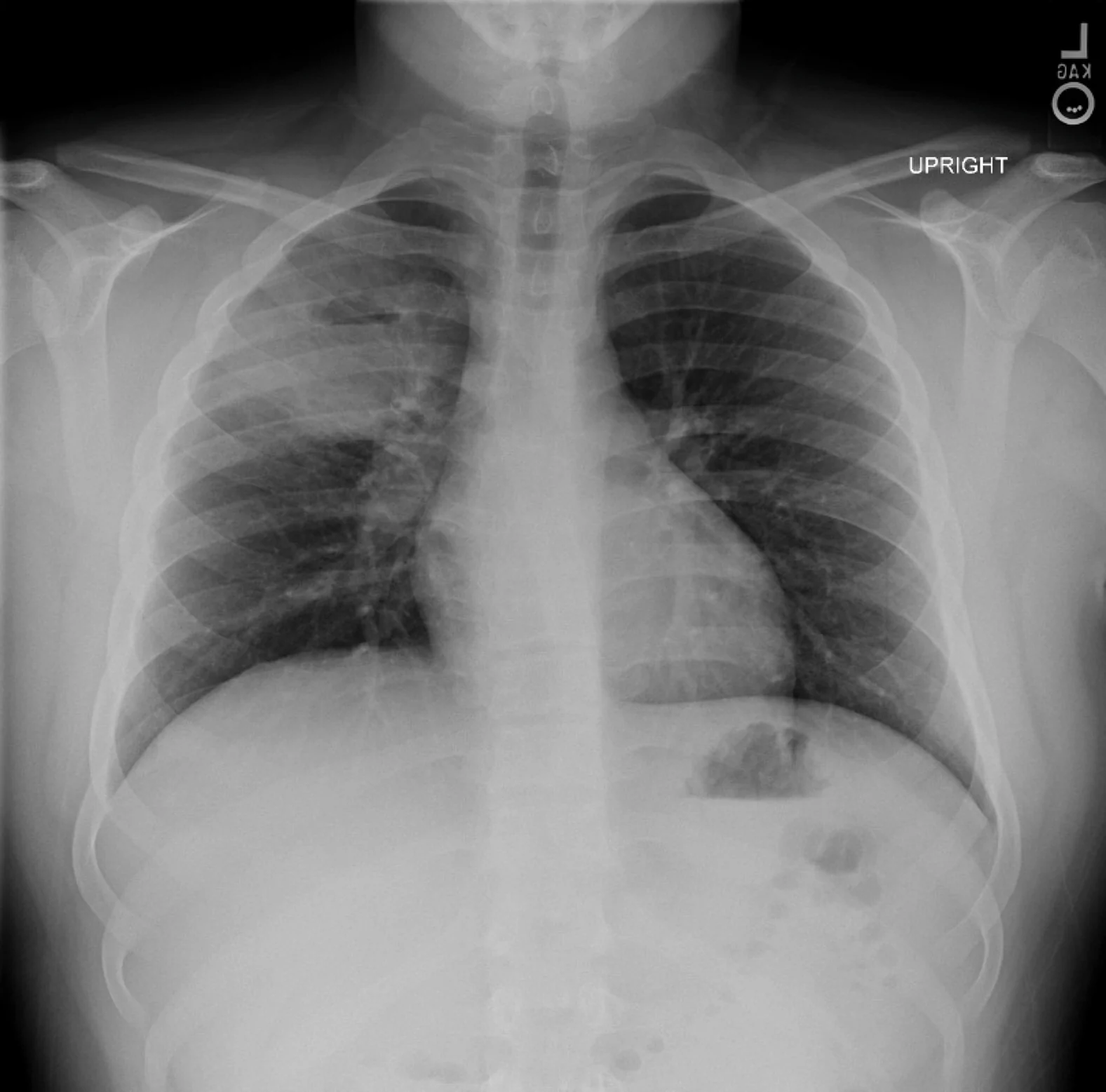
Anteroposterior chest radiograph demonstrating a right upper lobe round opacity with an initially unreported air-fluid level.

**Figure 2:**
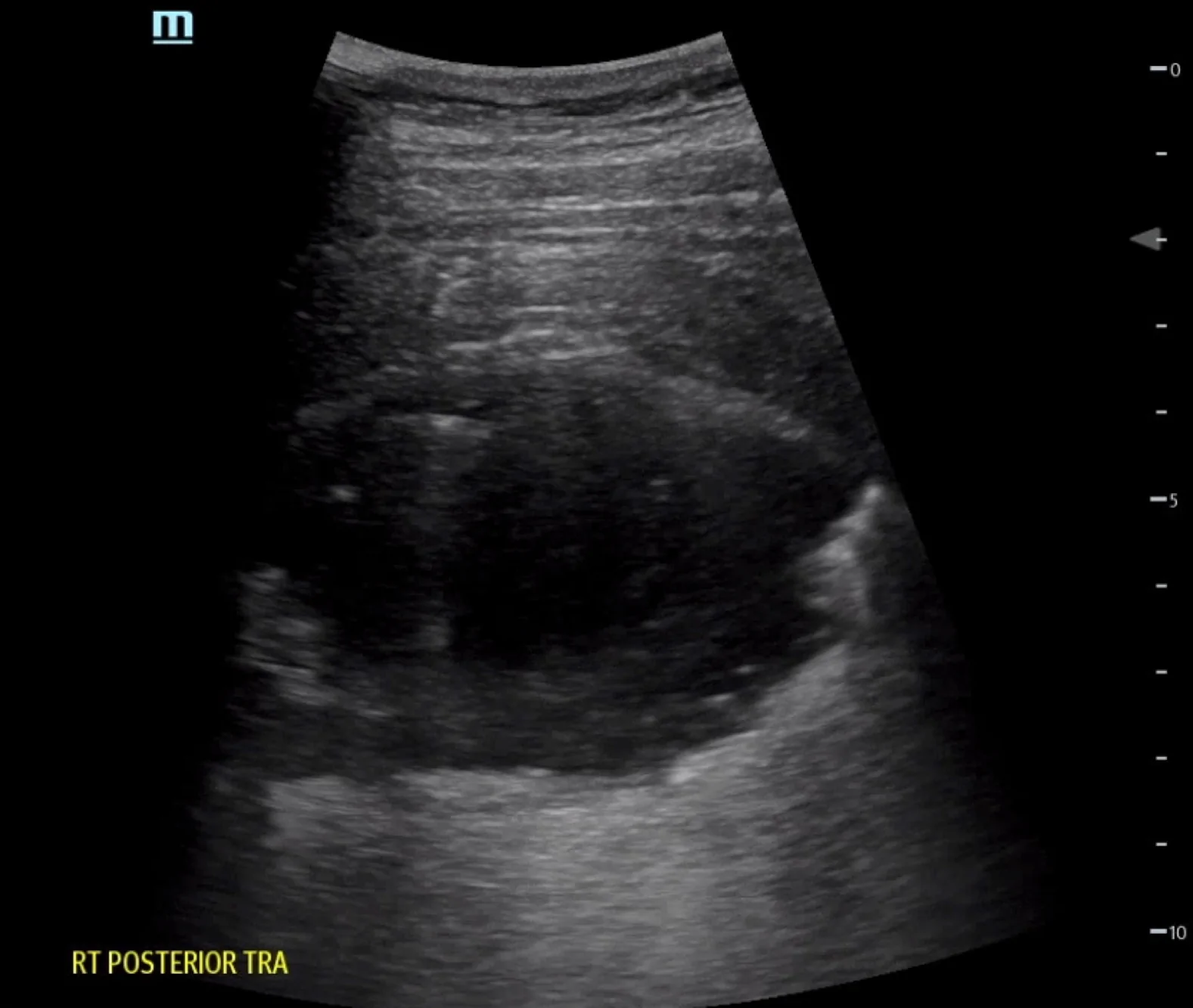
Transverse lung ultrasound of the right upper posterior lung field. An irregularly shaped, heterogeneous, hypoechoic lesion is visualized, demonstrating posterior acoustic enhancement and dynamic movement of internal echogenic material during respiration.

**Video 1:**
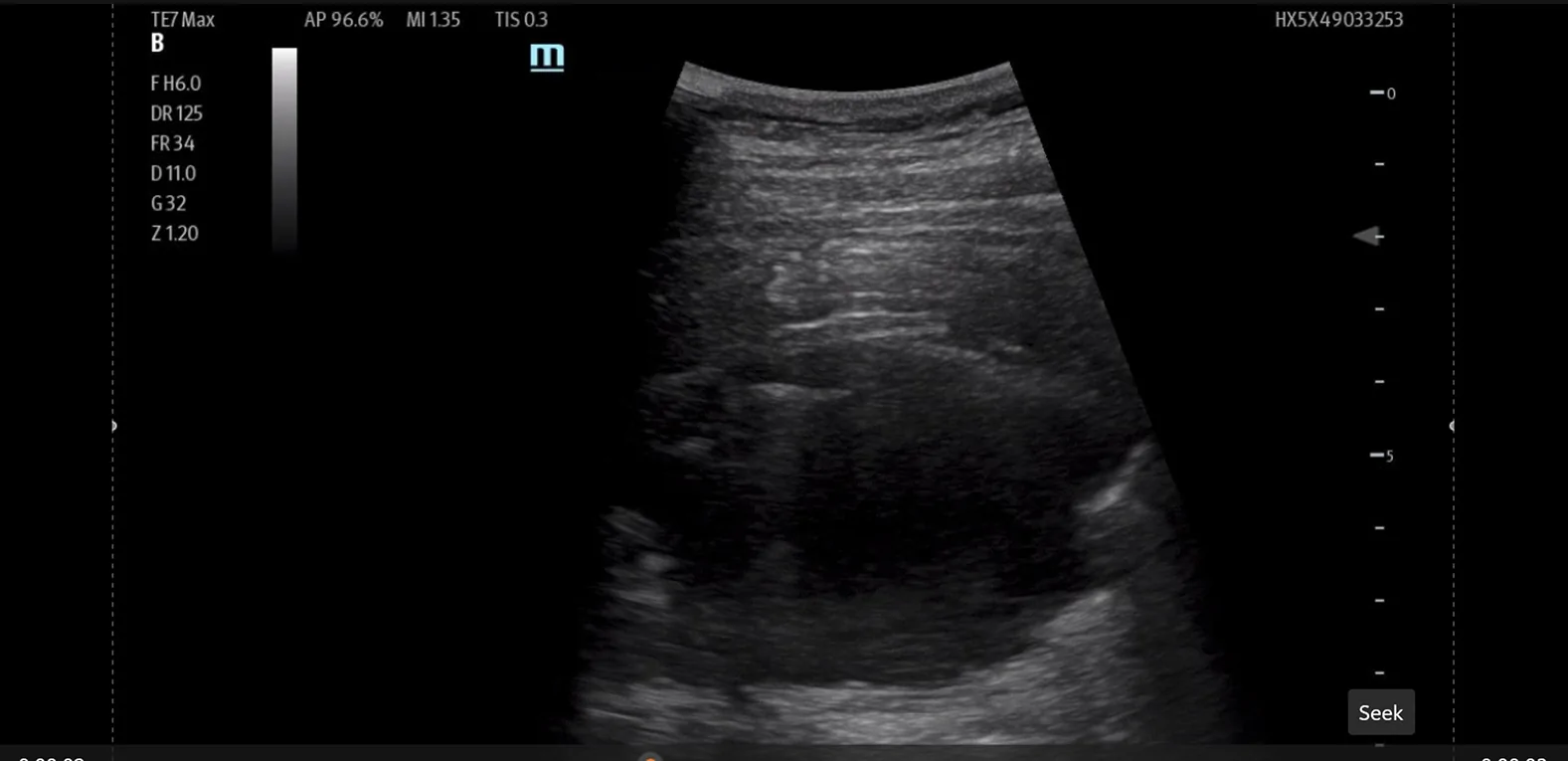
Transverse lung ultrasound of the right upper posterior lung field. An irregularly shaped, heterogeneous, hypoechoic lesion is visualized, demonstrating posterior acoustic enhancement and dynamic movement of internal echogenic material during respiration.

**Video 2:**
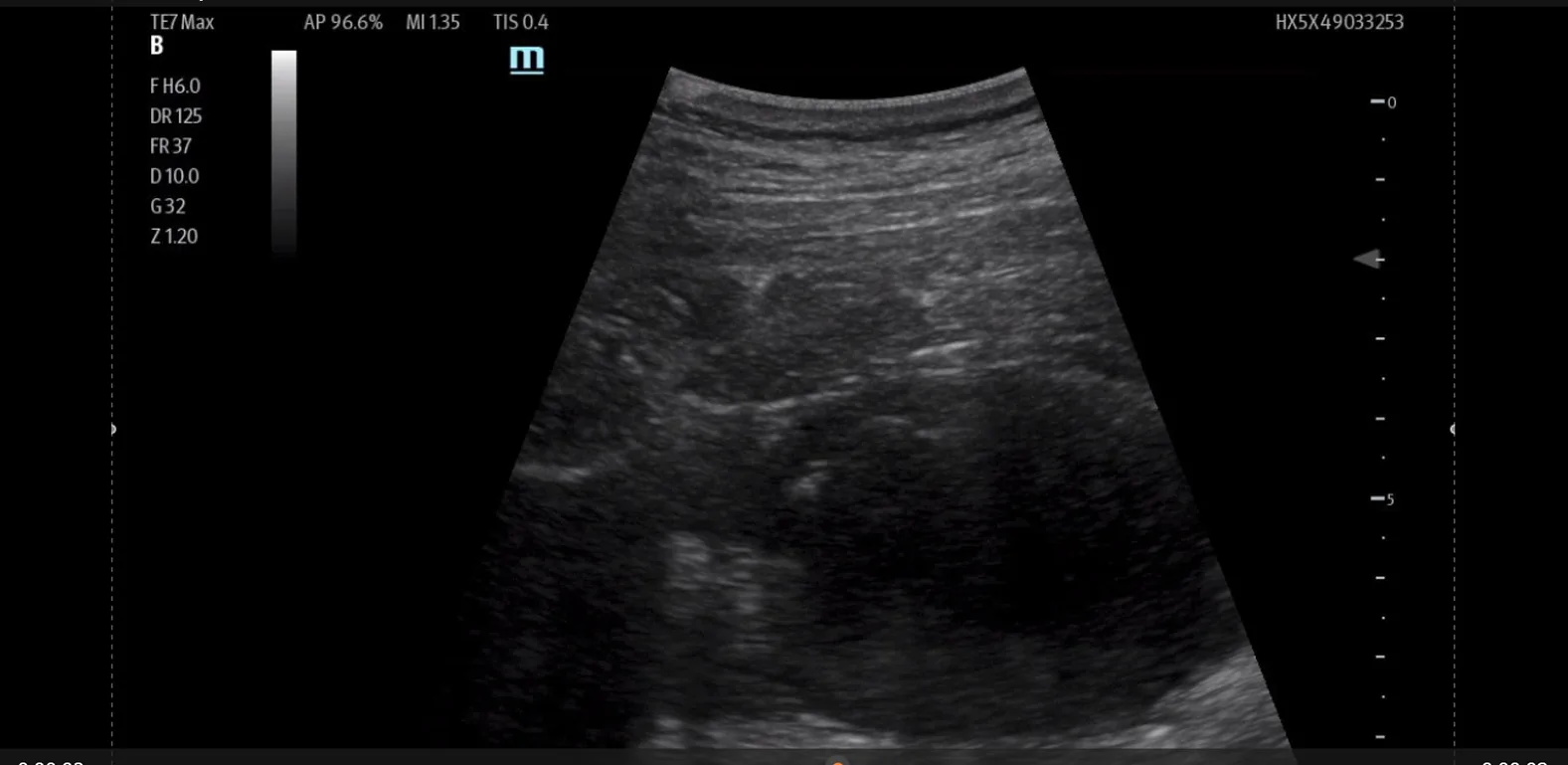
Transverse lung ultrasound of the right upper posterior lung field. Sequential scanning from superior to inferior reveals an irregularly shaped, heterogeneous, hypoechoic lesion demonstrating posterior acoustic enhancement.

**Figure 3:**
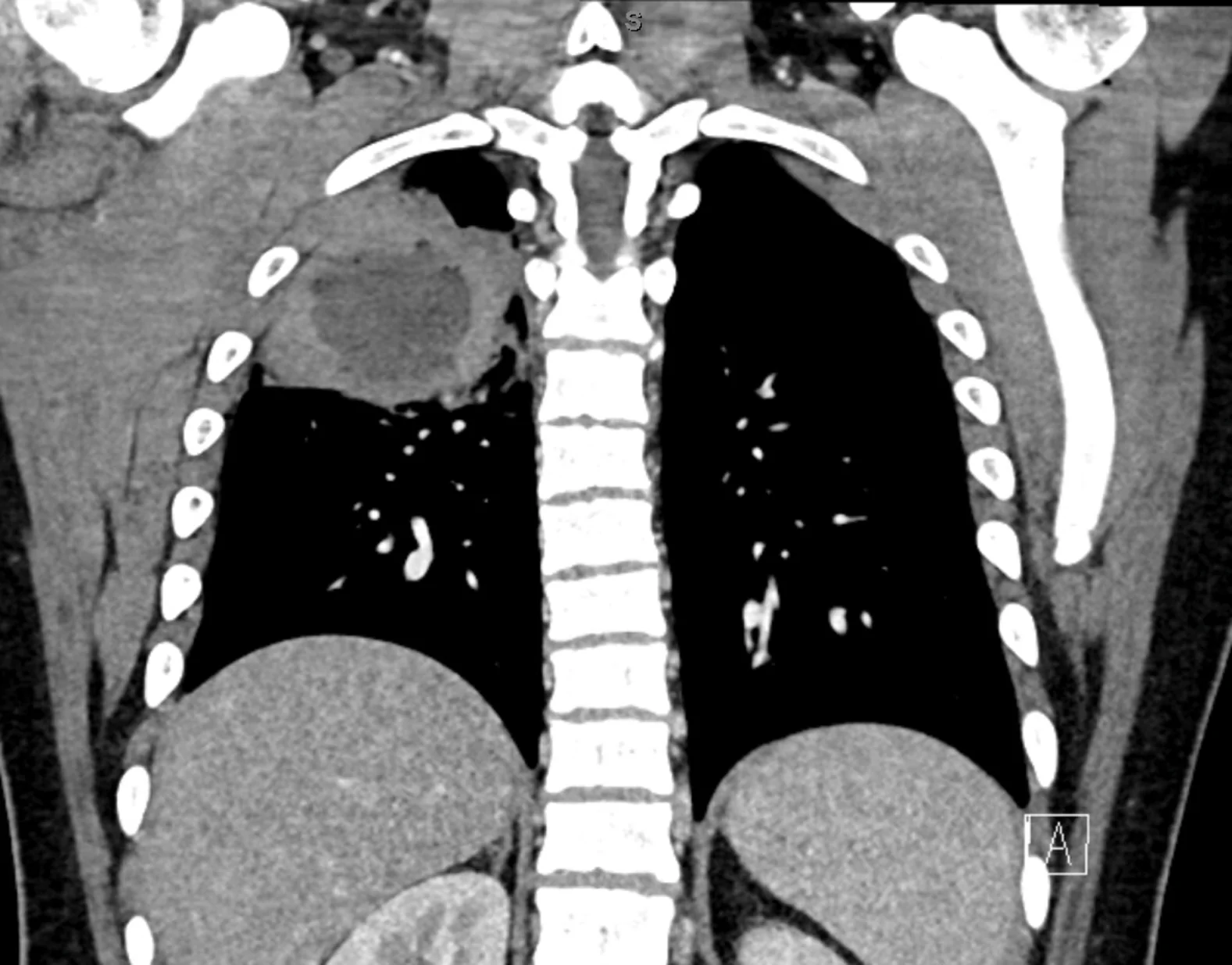
Coronal lung computed tomography scan demonstrating a 4.8-cm cavitary lesion in the right upper lobe, highly compatible with a pulmonary abscess.

## 2 Diagnosis: Pediatric Lung Abscess

Pediatric lung abscesses are rare, with an annual incidence of 0.7 cases per 100,000 admissions,^1^ and they can radiographically mimic round pneumonia. However, round pneumonia is uncommon in adolescents; Kim et al^2^ reported that 75% and 90% of cases occurred in children aged <8 and <12 years, respectively, because of the eventual development of collateral ventilation pathways and increased alveolar size.

While CT imaging is reserved for surgical planning, lung point-of-care ultrasound serves as a rapid, radiation-free diagnostic adjunct. Classic sonographic hallmarks—including irregular wall thickness, blurred outer margins, internal fluid-debris layers, and the absence of a split pleura sign^3^—allow emergency physicians to immediately detect necrotic parenchymal complications at the bedside, optimizing early clinical management and antibiotic selection.

## Funding and Support

By *JACEP Open* policy, all authors are required to disclose any and all commercial, financial, and other relationships in any way related to the subject of this article as per ICMJE conflict of interest guidelines (see www.icmje.org). The authors have stated that no such relationships exist.

## Ethics declaration

Written informed consent to take part in the study and to publish the article has been obtained from all participants or their legal representatives. The privacy rights of participants have been observed.

This study was performed in compliance with relevant laws, regulatory frameworks and guidelines where the research took place. Ethics committee approval was not required under relevant laws and institutional guidelines. This manuscript describes the clinical course of a single patient and does not meet the federal or institutional definition of systematic human subjects research. Therefore, formal Institutional Review Board (IRB) review and approval was not required.

## Conflict of Interest

All authors have affirmed they have no conflicts of interest to declare.
